# Human Commercial Models’ Eye Colour Shows Negative Frequency-Dependent Selection

**DOI:** 10.1371/journal.pone.0168458

**Published:** 2016-12-22

**Authors:** Isabela Rodrigues Nogueira Forti, Robert John Young

**Affiliations:** School of Environment and Life Sciences, Peel Building, University of Salford Manchester, Salford, United Kingdom; Monash University, AUSTRALIA

## Abstract

In this study we investigated the eye colour of human commercial models registered in the UK (400 female and 400 male) and Brazil (400 female and 400 male) to test the hypothesis that model eye colour frequency was the result of negative frequency-dependent selection. The eye colours of the models were classified as: blue, brown or intermediate. Chi-square analyses of data for countries separated by sex showed that in the United Kingdom brown eyes and intermediate colours were significantly more frequent than expected in comparison to the general United Kingdom population (P<0.001). In Brazil, the most frequent eye colour brown was significantly less frequent than expected in comparison to the general Brazilian population. These results support the hypothesis that model eye colour is the result of negative frequency-dependent selection. This could be the result of people using eye colour as a marker of genetic diversity and finding rarer eye colours more attractive because of the potential advantage more genetically diverse offspring that could result from such a choice. Eye colour may be important because in comparison to many other physical traits (e.g., hair colour) it is hard to modify, hide or disguise, and it is highly polymorphic.

## Introduction

It has been proposed that the variation observed in human eye colour is the result of sexual selection [1]. The diversity of human eye is thought to be the result of selection for rarity or in genetic terms negative frequency dependent selection, however, this hypothesis has not been tested by quantitative methods. Initially, everyone had brown eyes, and this situation lasted at least until modern humans entered northern Europe some 30,000 years ago. Analysis of DNA from skeletal remains has revealed that blue eyes were already present in humans at Motala, Sweden some 8,000 years ago [2]. Eye colour is the result of an expression of a number of different alleles and as such different phenotypes represent different allelic combinations and the system controlling eye colour expression although not fully understood is known to be highly polymorphic [1,3]. Although a wide range of human eye colours exist they are grouped together in three broad categories brown, blue and intermediate (e.g., green). It is also known that eye colour does not follow simple Mendelian rules of inheritance due to the action of several genes producing the observed phenotypes [4].

If blue eye colour indicates a genetic advantage, be it through genetic dissimilarity or other means, why has it not gone to fixation in northern European populations? [1]. There is extensive literature on guppies, lizards, insects and humans that shows a rarity advantage in terms of being selected as a mate [5]. At least one study has proven the link between phenotypic rarity and fitness [5], however, many cases exist where no such link has been shown. In these cases rarity could be maintained by negative frequency-dependent selection; that is, an individual is favoured for no other reason that being infrequent in the population. It should be noted that in these cases the rarity being selected for is strikingly different from existing phenotypes (e.g. human eyes can be sky blue but are not navy blue). Thus, individuals are choosing rare phenotypes to increase the genetic diversity of their offspring, which may provide their offspring with fitness advantages (1,5,6).

It is not just genetically determined traits that have been shown to be the result of negative frequency dependent selection, but also in human modifiable traits such as beards in men. Studies have shown that men with beards are rated as more attractive when beards are rare [5] and the same effect has been observed with the hair colour of women [6]. Thus cultural and genetic traits in humans may be maintained by negative frequency-dependent selection. However, in the aforementioned study with beards clean shaven men were not rated as more attractive when rare. Furthermore hair colour in women can be easily changed through dyes [7]. Although cosmetics and indeed cosmetic surgery allows people to modify certain physical traits to appear more attractive and rarer–modification of human eye colour through the use of coloured contact lenses has only occurred in the last 10 years. Thus, eye colour phenotypes have and continue to be reliable indicators of genetic diversity (i.e., polymorphisms).

How important are eyes in human mate choice? Certainly in romantic literature they are often mentioned, but in practice how much attention do people pay to the eyes of other individuals? Eye tracking studies show that when people first look at the faces of other people they focus much of their attention on the eyes of both real people and images of people [8]. In ‘Lonely Hearts Adverts’ the colour of someone’s eyes is one of the most cited physical characteristics and many people state they find one eye colour more attractive than others [9].

Interestingly eye colour in humans has importance beyond aesthetics or beauty. Eye colour in men is associated with how dominant they look: blue eyed men have more feminine face shapes than dark eyed men, which makes them look less dominant and this seems to be related to exposure to testosterone in the womb. Dominance is known to be an attractive trait to women [10]. Some studies have reported an apparent imprinting effect of eye colour with some people significantly preferring the eye colour of their opposite sex parent [11]. One study suggested blue eyed men prefer blue eyed women as this provides a visual mechanism for confirming paternity [12]. Furthermore eye colour variation has been found to be greater in women than men [1]. Recent studies have implicated eye colour has having a role in information processing (quicker by dark eyed people), sporting performance (dark eyed people being better at reactive sports), susceptibility to alcohol abuse (light eyed people being more susceptible), pain tolerance (light eyed people being more pain tolerant) and even temperament in young boys (blue eyed boys tending to be more shy than dark eyed boys, for example) [13,14,15,16,17,18]. It could therefore be argued that eye colour is not just an aesthetic characteristic indicating genetic diversity.

The problem of measuring eye colour attractiveness in the general population is that it is confounded by other measures of beauty such as facial symmetry. One solution to this problem is to use members of human society, which the majority of people find attractive; that is, commercial models [19]. It should be remember that commercial models represent human ideals in beauty (i.e., what people aspire to in terms of attractiveness in a partner) and not necessarily the choices people make–as these decisions are often compromises that take into account many factors (e.g., the person’s age, social status, wealth). Furthermore, commercial models are not subject to fashion selecting models of an extreme type as being attractive.

We chose to investigate whether the variation of human eye colour could be being maintained by negative frequency-dependent selection. We did this by comparing commercial models in two populations where eye colour frequencies were inverted. In the United Kingdom blue eyes make up more than 70 percent of the population [20], whereas in Brazil brown eyes account for more than 70 percent of the population [21]. Therefore we predicted that there would be significantly more brown eyed models than expected by chance in the UK population, and significantly more blue eyed models than expected by chance in the Brazilian population. Furthermore, we predicted that these effects will be less strong in male models due to the association between brown eyes and dominance, and that female models will show a greater variation in eye colour than male models.

## Material and Methods

Data were collected on the eye colour of human commercial models from the, publically accessible, on-line sites of model agencies in the United Kingdom (Select Model Management, Elite Models, Models 1 and Storm Management) and in Brazil (Mega Models, L’equipe Agence, Ford Models, Ten Model Management and Joy Management). We chose to use the data on commercial models and not fashion models for the following reasons: (1) these types of models are chosen for their physical attractiveness to the general population; (2) model agencies carefully classify the eye colour of their models due to the demands/requirements of clients; and (3) commercial models work for local clients in the country where they are registered. Thus, this dataset consists of people considered universally attractive, whose eye colour has been well defined and work in the geographic location of their agency. On the site of each agency the profile of models was presented in alphabetic order, and we recorded the eye colour of the first 100 female and 100 male models for each agency. We limited our sample size to 100 per agency to avoid any one agency having an undue influence on the dataset. It should be noted that agencies will use several ‘scouts’ or ‘bookers’ to recruit models (in this dataset at least 8 different individuals), who do so based on their perception of the market [12]. Furthermore, we were careful to avoid the counting of a model more than once (i.e., model registered at more than one agency). In the case of one Brazilian agency they did not have 100 male models; this problem was solved by including additional models from another agency, using the aforementioned rules, to bring the number of models up to 100. Thus, we collected data on 400 British female, 400 British male, 400 Brazilian female and 400 Brazilian male models. No filtering of models in terms of ethnicity or any other characteristic was undertaken; this was done because both the United Kingdom and Brazil have been multi-ethnic societies for centuries. The vast majority of models used were aged between 18 and 30 years old. No ethical approval was required for this research as we only used publically available data and no specific individuals have been identified.

The eye colours of models recorded was quite diverse from black, various shades of brown, blue mixtures, green and others (10 phenotypes in total). However, accurate data on eye colour frequency in the United Kingdom classifies eye colours as brown (all shades of brown and black), blue (all mixtures and grey) and other (mainly green) [20]. Therefore to allow comparisons between United Kingdom based and Brazilian based models eye colour was classified into one of the three aforementioned categories using United Kingdom based definitions [20], which were originally developed for forensic eye colour classification. This also had the advantage of removing any ambiguity (i.e. subjectivity) in eye colour definition.

It is known that eye colours varies broadly depending on the concentration of melanin pigment deposited in the iris as it is demonstrated with higher concentration in dark eyes or not present in blue eyes. This gradient of possible colours difficulties scientists’ categorization and gives them a certain degree of arbitrariness, especially when the amount of pigment falls between the two extremes colours as it happens with green or grey eyes [22]. Even though modelling agencies might not use the same criteria of academia for defining eye colours concerning the amount of melanin in the iris, we believe that it is still valid to use the information provided by their website since it is possible to separate their models into the three different phenotypes (brown, blue and intermediates), and this largely eliminates the problems arising from different interpretation of eye colours.

The data were analysed as the frequency of different eye colours by different countries and sexes using chi-square tests and the standardised residuals of each table cell. Thus, four chi-square tests were made. Expected frequencies for British and Brazilian models were derived from published data [20,21].

## Results

The eye colour frequencies of United Kingdom based models for both females and males was significantly different from that of the general United Kingdom population (Female χ^2^ = 182.00; d.f. = 2; *p*<0.001; Male: χ^2^ = 64.75; d.f. = 2; *p*<0.001). An analysis of the standardised residuals (Table 1) shows that blue eyes were significantly less frequent in the models than in the general United Kingdom population; whereas brown and other eye colours were significantly more frequent than in the general United Kingdom population for both sexes.

**Table 1:** Observed and expected eye colour frequencies in United Kingdom registered commercial models plus standardised residuals of a chi-square analysis for each sex.

Eye Colour	Male Observed Frequency	Female Observed Frequency	Expected Frequency^a^	Male Standardised Residual	Female Standardised Residual
Blue	214	187	286	-5.26***	-7.12***
Brown	132	120	84	3.82**	2.92**
Other	54	93	30	2.77**	6.18***

^a^data taken from Walsh et al. [20]

**p<0.01

***p<0.001

The eye colour frequencies of Brazil based models for both sexes was significantly different from that of the general Brazilian population (Female χ^2^ = 109.97; d.f. = 2; *p*<0.001; Male: χ^2^ = 92.42; d.f. = 2; *p*<0.001). An analysis of the standardised residuals (Table 2) showed that brown eyes were significantly less frequent in the models than in the general Brazilian population; whereas blue and other eye colours were significantly more frequent than in the general Brazilian population for both sexes.

**Table 2:** Observed and expected eye colour frequencies in Brazil registered commercial models plus standardised residuals of a chi-square analysis for each sex.

Eye Colour	Male Observed Frequency	Female Observed Frequency	Expected Frequency^a^	Male Standardised Residual	Female Standardised Residual
Blue	75	76	32	4.47***	4.55***
Brown	220	210	296	-5.62***	-6.31***
Other	105	114	72	2.81**	3.52**

^a^data taken from Ruiz-Linares et al. [21]

**p<0.01

***p<0.001

In Tables 1 and 2 it can be observed that male models had a greater frequencies of brown eyes than female models who in both cases had greater frequencies of other eye colours.

## Discussion

For both sexes in both countries rarer eye colours were significantly more frequent in commercial model populations than in the general population of the countries concerned. These data support the hypothesis that negative frequency dependent selection is operating on eye colour of commercial models; that is, rarer eye colours are being favoured. Furthermore the data in Tables 1 and 2 show a slight trend for brown eyes to be more common in males, and female models to display a greater diversity of eye colour.

These data provide quantitative support for the first time for the hypothesis that eye colour diversity in humans has been maintained by negative frequency-dependent selection [1]. Thus, human attraction to eye colour is based on a colour’s rarity in the population and not on it being a specific colour. The present data thus lend support to Frost’s speculation that eye colour diversity has been maintained by sexual selection. Furthermore, these data answer the question as to why eye colours have not gone to fixation in different populations. However, our data do not answer whether the previously discussed traits (e.g., information processing speed, sporting performance, susceptibility to alcohol abuse and pain tolerance [13,14]) associated with eye colour have influence consciously or subconsciously the selection of commercial models.

The data also showed that diversity of eye colours was greater in female than male models as would be predicted if sexual selection was acting more strongly on this sex; however, the size of this effect was small. Male models had higher frequency of brown eyes in both populations but the differences were minimal. The investigation of these effects probably requires much larger sample sizes to produce more definitive answers.

It is interesting that in humans fashion is based on a ‘unique look’ but once that ‘look’ becomes common in the population selection acts against it [5,6,19]. Given that the ability to modify eye colour only occurred in the last three decades [23], this may explain human fascination with this physical characteristic; whereas humans have been able to modify bodily traits through the use of cosmetics for approximately 100,000 years [24]. Thus, eye colour may be perceived as an honest signal of genetic rarity. Overall this study helps to explains how genetic or cultural selection can result in increasing diversity or cyclic variation in phenotypically rare traits [1,5,19].
